# Coblation Versus Radiofrequency for Tongue Base Reduction in Obstructive Sleep Apnea: A Meta‐analysis

**DOI:** 10.1002/oto2.70076

**Published:** 2025-01-19

**Authors:** Salman Hussain, Jafar Hayat, Raisa Chowdhury, Mahmoud Ebrahim, Abdulmohsen Alterki, Ahmed Bahgat, Ahmed A. Al‐Sayed, Vikram Padhye, Robson Capasso

**Affiliations:** ^1^ Department of Otolaryngology–Head and Neck Surgery University of Ottawa Ottawa Ontario Canada; ^2^ Department of Otolaryngology–Head and Neck Surgery Jaber Al‐Ahmad Hospital Kuwait Kuwait; ^3^ Faculty of Medicine and Health Sciences McGill University Montreal Quebec Canada; ^4^ Department of Otolaryngology–Head and Neck Surgery McGill University Montreal Quebec Canada; ^5^ Department of Otolaryngology–Head and Neck Surgery Zain Hospital Kuwait Kuwait; ^6^ Medical Department Dasman Diabetes Institute Dasman Kuwait; ^7^ Department of Otolaryngology–Head and Neck Surgery Alexandria University Alexandria Egypt; ^8^ Department of Otolaryngology–Head and Neck Surgery, College of Medicine King Saud University Riyadh Saudi Arabia; ^9^ Department of Otolaryngology–Head and Neck Surgery, School of Medicine, Division of Sleep Surgery Stanford University Stanford California USA

**Keywords:** ablation, channeling, coblation, obstructive sleep apnea, OSA, radiofrequency, resection

## Abstract

**Objective:**

The objective of this study is to determine the effectiveness and safety profile of coblation tongue base reduction (CBTR) compared to radiofrequency base of tongue (RFBOT) reduction on sleep‐related outcomes in patients with obstructive sleep apnea (OSA).

**Data Sources:**

PubMed, Scopus, Web of Science, and Cochrane Database of Systematic Reviews databases.

**Review Methods:**

Literature search by 2 independent authors was conducted using the abovementioned databases. Studies on CBTR and RFBOT as part of OSA treatment in adult patients with pre‐ and postoperative apnea–hypopnea index (AHI) were included. Direct meta‐analysis and single‐arm meta‐analysis were conducted to compare sleep‐related outcomes (AHI, apnea index [AI], surgical success rates, Epworth sleepiness score [ESS], SpO_2_, body mass index [BMI], and visual analog scale [VAS]) between both groups.

**Results:**

A total of 40 studies with a total of 1940 patients were included, of which 1440 individuals who underwent tongue base reduction interventions (RF = 306, RF + UPPP = 656, and coblation + UPPP = 482) met inclusion criteria. Pooled analysis showed significant improvements in AHI (CBTR = −22.84, RFBOT = −11.14), AI (CBTR = 15.64, RFBOT = −5.26), ESS (CBTR = −7.59, RFBOT = −7.18), mean oxygen saturation (CBTR = 7.43, RFBOT = 4.25), mean BMI (CBTR = −0.69, RFBOT = −4.09), and snoring visual analog scale (CBTR = −16.20, RFBOT = −18.21). Surgical success rate (postoperative AHI < 20 and drop >50% from baselines) was 70% for CBTR and 43% for RFBOT.

**Conclusion:**

Both interventions decreased sleep‐related outcomes in adult patients with OSA. Coblation appears to exhibit superiority over radiofrequency with a similar safety profile. However, further studies with direct comparisons between both interventions must be performed.

Obstructive sleep apnea (OSA) is characterized by repeated episodes of complete (apnea) or partial (hypopnea) cessation of respiratory passages during sleep resulting in sleep fragmentation and repeated arousals due to nocturnal hypoxemia.^1^ OSA is an increasingly prevalent disorder affecting 9% to 38% of the general adult population.^2^ It is emerging as a major health problem, with a high disease burden due to the health care costs associated with OSA and due to its association with significant implications on long‐term health and quality of life as it has been consistently shown to be an independent risk factor for cardiopulmonary diseases and metabolic and neurobehavioral disorders.^3^

Continuous positive airway pressure (CPAP) is considered as the gold standard of care for moderate to severe OSA, but because of the high rates of CPAP nontolerance and noncompliance, surgical treatment has become a viable OSA treatment option.^4^ The surgical management for OSA is directed toward the anatomical target of interest, and these areas typically overlap in a single patient; therefore, surgical management usually involves a multilevel approach.^5^ Oropharyngeal and retroglossal airway obstructions are commonly identified in patients with OSA; therefore, the base of tongue (BOT) represents an anatomical target of interest.^6^ A substantial portion of the obstruction during sleep is caused by obstruction at the base of the tongue, which affects about 70% of subjects.^1^ Various surgical procedures have been developed to address obstruction of the tongue base, such as tissue repositioning (tongue suspension, genioglossal advancement, and maxillomandibular advancement) and tissue debulking procedures (midline glossectomy and radiofrequency ablation), and each procedure has a different outcome.^7^, ^8^, ^9^, ^10^, ^11^, ^12^ However, because of the difficult surgical access and visualization, as well as the uncertainty surrounding the volume of tissue that can be safely excised, tongue base surgery remains challenging.

Sher et al demonstrated in their meta‐analysis of 37 included studies that, in patients with retrolingual collapse, the success rate of uvulopalatopharyngoplasty (UPPP) alone was 5% to 10%.^13^ This finding has been consolidated by additional studies concluding that untreated tongue base hypertrophy is the main cause of surgical treatment failure in OSA.^14^ Therefore, BOT is an anatomical area that should not be overlooked by sleep surgeons. To address BOT hypertrophy, radiofrequency base of tongue (RFBOT) reduction and coblation tongue base reduction (CTBR) are 2 commonly employed interventions, and both are considered viable and effective surgical options for BOT hypertrophy.^4^ Several studies have been published previously in literature providing evidence of their effectiveness, but very few studies comparing the 2 techniques. The aim of the current systematic review and meta‐analysis is to evaluate the effectiveness and safety profile of RFBOT and CTBR for tongue base reduction in patients with OSA and provide recommendations for future research.

## Methods

### Study Protocol

This systematic review and meta‐analysis was based on a prespecified protocol. The Cochrane Handbook for Systematic Reviews of Interventions and the Preferred Reporting Items for Systematic Reviews and Meta‐analyses (PRISMA) statement were followed during the preparation of this research.

### Eligibility Criteria and Study Selection

The inclusion criteria comprised the following: (i) patients: adults >18 years of age diagnosed with OSA who underwent coblation or RFBOT ablation, resection, or channeling, either alone or as part of multilevel sleep surgery; (ii) intervention: coblation ablation or resection; (iii) comparator: radiofrequency ablation or channeling; (iv) study outcomes: primary outcomes were surgical success rate as defined by Sher's criteria (postoperative AHI < 20 and drop >50% from baseline), mean reduction in apnea–hypopnea index (AHI) postoperatively, mean reduction in apnea index (AI) postoperatively, and Epworth sleepiness scale score. Secondary outcomes included mean change in oxygen saturation postoperatively, mean change in body mass index (BMI) postoperatively, mean change in snoring postoperatively as defined by visual analog scale (VAS), and postoperative complications. (v) Study design: randomized‐controlled trials (RCTs), observational studies, and prospective and retrospective cohort studies.

The exclusion criteria comprised the following: (i) studies of unrelated diagnosis, (ii) studies involving in vitro studies; and (iii) studies other than RCTs and prospective and retrospective cohort studies such as case reports, review articles, and letters.

### Information Sources, Search Strategy, and Study Selection

PubMed, Scopus, Web of Science, and Cochrane Database of Systematic Reviews databases were systematically screened from inception until January 2024 by 2 authors (S.H. and J.H.). The search strategy utilized the search terms “coblation,” “radiofrequncy,” “low temperature radiofrequency,” “low temperature bipolar radiofrequency,” “tongue base reduction,” “tongue reduction,” “tongue base resection,” “base of tongue reduction,” “obstructive sleep apnea,” “obstructive sleep apnea hypopnea syndrome,” “OSA,” and “OSAHS.” To broaden the literature search, we scanned the reference lists of eligible studies and contemporary reviews for potentially missed relevant studies. The study selection process comprised omitting duplicate citations, followed by screening of titles and abstracts for possible inclusion by 2 reviewers (S.H. and J.H.) independently to identify articles for full‐text review. Articles were only included if both reviewers independently determined that all inclusion criteria were met. If consensus was not reached, a third author (M.E.) was consulted.

### Quality Assessment of the Included Studies

Two authors (S.H. and J.H.) assessed the quality of clinical trials using the Cochrane Risk of Bias 2 tool (ROB‐2) for RCTs, which covers 5 domains: randomization, deviation, outcome, missing data, and reporting. The risk of bias was categorized as low, high, or some concerns. Disagreements among the authors were resolved by consulting a third author (M.E.). Quality of observational studies was completed using the Newcastle Ottawa scale (NOS), which evaluates selection, comparability, and outcome. The NOS score ranges from 0 to 9 stars, with higher scores indicating lower bias. We considered studies with 7 or more stars as low bias, 4 to 6 stars as moderate bias, and 3 or less stars as high bias.

For nonrandomized studies of interventions (NRSI), we used ROBINS‐I tool. We specified the important confounding domains and co‐interventions of concern for each outcome in our protocol. We assessed the risk of bias in 7 domains: confounding, selection of participants, classification of interventions, deviations from intended interventions, missing data, measurement of outcomes, and selection of the reported result. We answered signaling questions for each domain and made judgments of low, moderate, serious, or critical risk of bias.

Of the included studies, 9 were categorized as “good” quality, comprising 638 patients, with 5 being prospective and 4 being retrospective studies, with examples including Steward (29 patients), Blumen (11 patients), and Hou (40 patients), among others. Additionally, 7 studies were categorized as “fair” quality, comprising 295 patients, with 4 being prospective and 3 being retrospective studies, with notable studies such as Chen (22 patients) and Friedman (2007) (122 patients), among others.

### Data Collection and Study Endpoints

Two authors (S.H. and J.H.) collected a summary of the baseline characteristics of the included studies and participants, such as the first author's name and year of publication (study identifier), study arms, total sample size, age, gender, preoperative and postoperative BMI and neck circumference, method of addressing the BOT, the technique employed, and whether this was done alone or as part of multilevel sleep surgery.

Our endpoints included success rate as defined by Sher's criteria (postoperative AHI < 20 and drop >50% from baseline), mean reduction in AHI postoperatively, mean reduction in AI postoperatively, and ESS reduction. Secondary outcomes included mean change in oxygen saturation postoperatively, lowest oxygen saturation postoperatively, mean change in BMI postoperatively, mean change in snoring postoperatively as defined by VAS, and postoperative complications. Two coauthors (S.H. and J.H.) independently collected data using a predesigned extraction sheet, and discrepancies were settled by consultation with the principal investigator.

Studies included in the coblation + UPPP group underwent tongue base reduction with coblation (irrespective of technique employed) along with modified UPPP (variations in addressing soft palate included barbed reposition pharyngoplasty, expansion sphincter pharyngoplasty, z‐palatopharyngoplasty, and lateral pharyngoplasty). RF + UPPP involved radiofrequency ablation of BOT with modified UPPP as described in the coblation group. Groups that underwent RF alone referred to those patients who underwent RF channeling either under local anesthetic in an outpatient setting or under general anesthesia. Some studies in this group also underwent RF channeling of the soft palate. The specific number of passes used in coblation techniques was inconsistently reported, which may affect the comparability of efficacy outcomes. For coblation, various techniques were employed in the included studies. This includes coblation endoscopic lingual lightening (CELL) which is a variation of glossectomy to reduce tongue collapse, submucosal minimally invasive lingual excision (SMILE), and the RoboCob technique which uses principles from transoral robotic surgery but is instead carried out using the coblator wand. In some studies, large‐area ablation was primarily utilized to achieve adequate tongue tissue reduction via ablation.^15^, ^16^, ^17^

### Statistical Analysis

The data analysis was done utilizing the RevMan version (Version 5.3) and Rstudio (Version 4.2.2) for Microsoft Windows.

For the direct meta‐analysis part, mean difference (MD) was used as the effect size when assessing continuous variables. A 95% confidence interval (CI) was used, and a *P* value of <.05 was considered statistically significant. The random‐effects model was employed.

For the single‐arm meta‐analysis of continuous variables, it was done employing the “metamean” function from the “meta” package. The “metamean” function utilizes the method of moments to estimate the overall mean effect size across studies. The raw mean effect size (MRAW) was employed, and a random‐effects model was chosen to account for potential heterogeneity among studies. The restricted maximum likelihood (REML) estimator was utilized for estimating the between‐study variance (*τ* ^2^), and the Hartung–Knapp (HK) adjustment was applied to derive CIs for the random‐effects model.

In case of single‐arm meta‐analysis of dichotomous data, a random intercept logistic regression model was employed, employing maximum‐likelihood estimation for *τ* ^2^. The calculation of random‐effects CIs relied on the *t*‐distribution, and a logit transformation was applied accordingly.

To assess heterogeneity in both direct and single‐arm meta‐analyses, the *I* ^2^ statistic was used to quantify heterogeneity. *I* ^2^ = 50% with a *P* value of <.1 was considered as indicative of statistically significant heterogeneity. Egger's test was used to perform publication bias. If *P* < .005, then Egger's test is considered significant and that refers to the presence of publication bias.

## Results

### Summary of Literature Search

**Figure 1:**
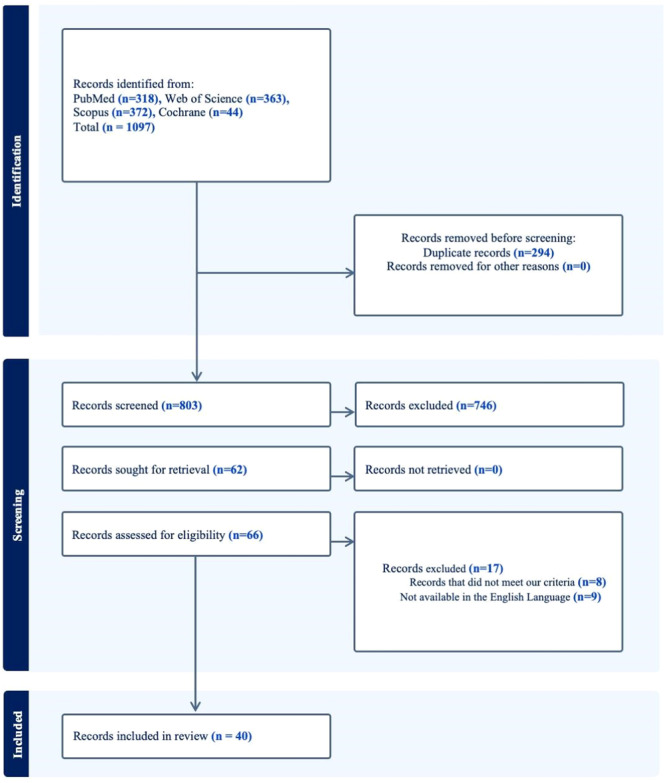
The Preferred Reporting Items for Systematic Reviews and Meta‐analyses (PRISMA) flowchart for literature search and study selection.

The database search yielded 1097 citations, of which 294 citations were omitted due to duplication. Of the remaining 803 citations, 746 were omitted during title/abstract screening, and the remaining 62 underwent full‐text screening, of which 17 were excluded. Finally, 40 studies were included in the systematic review. **Figure** 1 summarizes the PRISMA flowchart for literature search and study selection.^9^, ^10^, ^11^, ^12^, ^13^, ^14^, ^15^, ^16^, ^17^, ^18^, ^19^, ^20^, ^21^, ^22^, ^23^, ^24^, ^25^, ^26^, ^27^, ^28^, ^29^, ^30^, ^31^, ^32^, ^33^, ^34^, ^35^, ^36^, ^37^, ^38^, ^39^, ^40^, ^41^, ^42^, ^43^, ^44^, ^45^, ^46^, ^47^, ^48^, ^49^, ^50^

### Summary of the Characteristics of the Included Studies and Participants

In total, 1940 individuals underwent interventions for OSA, with 1440 of them receiving treatments specifically targeting tongue base hypertrophy across 40 studies (RF = 306, RF + UPPP = 656, and coblation + UPPP = 482). All research participants were adults, with an average age of 58.75 years. The gender distribution was predominantly male (male = 1515, female = 425), resulting in a male‐to‐female ratio of approximately 3.5:1. The average BMI across studies was 36.86. Reported preoperative AHI values varied widely, ranging from 5 ± 6 to 66.38 ± 17.03 events per hour. **Table** 1 provides a summary of the baseline characteristics of the included studies and participants.

**Table 1:** Baseline Characteristics of Included Studies and Participants

Study ID	Study arms	Number of patients (n)	Age (mean ± SD)	Gender (M/F)	Preop BMI (kg/m^2^) (mean ± SD)	Neck circumference (cm) (mean ±SD)	Surgical success rate by Sher criteria (n)	Preop AHI (mean ± SD)	Preop AI (mean ± SD)	Preop snoring (VAS scale) (mean ± SD)	Preop ESS (mean ± SD)	Preop SpO_2_ (mean ± SD)
Nelson et al (2001)	RF + UPPP	20	35.8	NA	NA	NA	4	47.2 ± 29.1	23.8 ± 24.2	4.92 ± 3.23	10.31 ± 4.85	NA
Stuck et al (2002)	RF	18	NA	14/4	28.9 ± 2.7	NA	NA	19 ± 11	5 ± 6	NA	12.9 ± 4.2	NA
Fischer et al (2003)	RF	15	56.0 ± 11.1	13/3	27.5 ± 22.8	NA	NA	NA	NA	7.5 ± 1.2	11.1 ± 4.7	NA
Friedman et al (2003)	UPPP only	134	40.2 ± 13.7	98 /36	30.0 ± 5.6	NA	NA	35.4 ± 25	10 ± 19.6	NA	NA	83.8 ± 12.5
	RF + UPPP	143	47.0 ± 11.7	104/39	31.5 ± 4.8	NA	NA	43.9 ± 23.7	10.1 ± 17.2	7.6 ± 1.1	15.2 ± 3.1	81.4 ± 10.4
Riley et al (2003)	RF	19	49.5 ± 10.7	15/4	30.0 ± 5.8	NA	10	NA	NA	NA	NA	NA
Woodson et al (2023)	Placebo	30	46.0 ± 8.1	21/9	28.5 ± 4.2	40.6 ± 3.6	NA	15.4 ± 7.8	3.9 ± 4.1	0.37 ± 0.78	11.6 ± 3.5	NA
	RF	29	49.4 ± 9.2	26/3	27.7 ± 3.6	40.9 ± 3.3	NA	21.3 ± 11.1	7.5 ± 10.9	0.64 ± 1.46	11.9 ± 4.6	NA
	CPAP	28	51.7 ± 8.6	22/6	29.1 ± 3.7	41.4 ± 3.3	NA	19.8 ± 9.9	6.2 ± 7.5	NA	12.6 ± 5	NA
Stuck et al (2004)	RF	18	49.69 ± 8.72	14/4	27.49 ± 2.81	NA	NA	NA	6.22 ± 6.63	7.82 ± 1.88	NA	NA
Steward et al (2005)	RF	29	49 ± 9	23/6	29 ± 4	41 ± 3	NA	NA	NA	7.5 ± 2.4	6 ± 3.7	NA
Blumen et al (2006)	RF	10	57.9 ± 8.9	NA	NA	NA	NA	NA	40.8 ± 21.6	6.2 ± 2.3	8.7 ± 5.6	64.2 ± 13
Herder et al (2006)	RF ± UPPP	22	47.4 ± 9.4	18/2	26.7 ± 2.8	NA	NA	35.1 ± 18.1	NA	1.2 ± 0.6	12.4 ± 2.9	82 ± 8.7
Richard et al (2006)	RF + UPPP	22	50.3 ± 7.7	20/2	27.7 ± 3.4	NA	NA	35.6 ± 9.2	NA	8.3 ± 1.6	14.2 ± 3.4	85.6 ± 3.4
Welt et al (2006)	RF	20	41.9 ± 8.1	15/5	26.2 ± 3.3	NA	10	9.3 ± 8.4	NA	NA	5.7 ± 5	NA
Bassiouny et al (2007)	RF	20	37.05	18/2	27.7 ± 3.8	NA	8	15.3	NA	7	NA	NA
	RF “cutting”	20	39.15	15/5	28.5 ± 3.2	NA	10	17.2	NA	7.5	NA	NA
Friedman et al (2007)	RF + UPPP	122	42.2 ± 11.4	80/42	28.3 ± 5.0	NA	54	23.2 ± 7.6	0.9 ± 2.9	9.4 ± 0.9	9.7 ± 3.9	88.9 ± 4.8
Tvinnereim et al (2007)	RF	40	46 ± 12	28/12	25.5 ± 7.2	NA	NA	NA	7.11 ± 7.68	7.4 ± 1.99	NA	NA
Eun et al (2008)	RF + UPPP	66	44.7 ± 10.6	58/8	27.6 ± 3.4	NA	30	22.9 ± 14.7	14.4 ± 11.5	8 ± 2.1	11.4 ± 5	79.1 ± 5.7
Friedman et al (2008)	RF + UPPP	48	46.1 ± 11.7	39/9	29.0 ± 4.4	NA	NA	38.9 ± 22.2	7.5 ± 16.8	9.5 ± 1	11.1 ± 3.1	85.4 ± 7.7
	Coblation (SMILE) + UPPP	48	44.0 ± 9.1	40/8	30.6 ± 5.2	NA	NA	44.5 ± 20	6.3 ± 12.3	9.5 ± 1.3	11.3 ± 3	84.6 ± 6.6
Ceylan et al (2009)	RF	26	46.3 ± 3.9	23/3	28.6 ± 3.8	40.4 ± 3.4	NA	29.6 ± 7.8	NA	NA	10.8 ± 3.2	86.8 ± 8.9
	CPAP	21	47.1 ± 4.1	19/2	29.1 ± 3.6	41.1 ± 3.2	NA	28.5 ± 6.9	NA	NA	11.1 ± 3.1	88.4 ± 8.5
Eun et al (2009)	RF + UPPP	28	46.5 ± 11.3	25/3	25.7 ± 2.7	37.9 + 7.2	14	14.5 ± 5.6	7.7 ± 6.1	8.3 ± 1.6	11.8 ± 4.6	78.2 ± 7.3
Neruntarat et al (2009)	RF	72	35.8 ± 10.9	69/3	28.8 ± 2.4	NA	5	29.5 ± 14.8	8.7 ± 6.4	7.27 ± 0.95	12.81 ± 2.68	NA
Babademez et al (2010)	Coblation + ESP	16	41.3 ± 10.5	16/0	29.6 ± 2.5	NA	10	20.1 ± 10.5	NA	NA	NA	84.6 ± 3.4
Babademez et al (2011)	Coblation (ablation) + UPPP	15	48.2 ± 7.1	40/15	NA	NA	NA	22.7 ± 12.3	NA	7.6 ± 1.7	8.4 ± 7.2	NA
	Coblation (SMILE) + UPPP	15	NA	NA	NA	NA	NA	17.7 ± 7.8	NA	8.4 ± 0.9	6.8 ± 3.7	NA
	Ultrasonic (harmonic scalpel) + UPPP	15	NA	NA	NA	NA	NA	29.9 ± 29.4	NA	8.4 ± 0.9	7 ± 3.7	NA
Hou et al (2012)	Coblation^a^	20	42.0 ± 8.4	NA	26.3 ± 2.9	NA	8	40.9 ± 10.4	NA	NA	NA	70.2 ± 11.6
	Coblation^b^	20	43.7 ± 11.8	NA	26.8 ± 3.2	NA	6	41.3 ± 9	NA	NA	NA	69.2 ± 12
Eun et al (2013)	UPPP	22	44.9 ± 13.1	22/0	NA	NA	NA	NA	NA	NA	NA	NA
	RF + UPPP	25	44.9 ± 13.1	25/0	NA	NA	NA	NA	NA	NA	NA	NA
MacKay et al (2013)	Coblation	47	41.1 ± 13.0	2/2	NA	NA	32	23.36 ± 20.06	NA	NA	9.8 ± 6.1	86 ± 4.5
MacKay et al (2013)	Coblation	4	53 ± 10.9	41/7	NA	NA	NA	39.15 ± 4.1	NA	6.25 ± 2.36	10.25 ± 4.57	84.25 ± 5.56
Plzak et al (2013)	RF + UPPP	79	50.5 ± 9.1	62/17	28.1 ± 3.1	NA	41	28.7 ± 17.1	12.1 ± 6.8	8.4 ± 1.9	10.6 ± 3.8	95.4 ± 3.9
	UPPP	35	47.9 ± 8.7	29/6	28.0 ± 3.3	NA	14	27.9 ± 14.1	11.9 ± 5.7	8.3 ± 2.1	10.1 ± 3.7	95.8 ± 1.9
Wang et al (2013)	Coblation + ZPPP	36	44 ± 9.75	31/5	29.2 ± 2.9	NA	NA	9.86 ± 4.81	0.742 ± 1.016	8.08 ± 0.86	10.5 ± 2.7	NA
Li et al (2015)	Coblation + UPPP	25	42 ± 9	20/5	26.5 ± 3.0	NA	18	45.7 ± 21.7	12 ± 13	NA	9.6 ± 4.9	77.1 ± 10.5
Lin et al (2015)	Coblation + ZPPP	35	42	32/3	27.2 ± 2.7	NA	22	50.6 ± 16.6	NA	9.86 ± 0.69	11 ± 4.2	70.4 ± 9.9
Li et al (2016)	Coblation (CELL) + UPPP	30	41.5 ± 9.4	27/3	26.4 ± 3.0	NA	NA	48.4 ± 16.9	16.8 ± 14.2	NA	10.9 ± 4.7	76.4 ± 8.5
	UPPP	60	40.3 ± 9.2	59/1	26.6 ± 2.8	NA	NA	44.2 ± 19.3	16.2 ± 19.1	NA	11.4 ± 5	77.5 ± 7.9
Chen et al (2019)	Coblation + ZPPP total	22	NA	20/2	NA	NA	NA	66.38 ± 17.03	57.96 ± 19.73	NA	NA	61.89 ± 12.54
Bahgat et al (2020)	Coblation (CELL) + UPPP	25	42.36 ± 9.08	NA	31.28 ± 2.96	NA	NA	36.96 ± 10.43	NA	NA	NA	NA
	Coblation (resection) + UPPP	25	41.36 ± 8.72	NA	30.48 ± 3.88	NA	NA	33.84 ± 10.54	NA	NA	NA	NA
Bahgat et al (2020)	Coblation (CELL) + UPPP	25	41.36 ± 8.72	17/8	30.48 ± 3.88	NA	NA	33.84 ± 10.54	NA	NA	NA	NA
MacKay et al (2020)	RF + UPPP	51	42.7 ± 12.8	41/10	30.7 ± 3.98	41.4 ± 3.8	13	47.9 ± 23.1	12.7 ± 14.5	NA	12.4 ± 3.6	79 ± 8.7
	CPAP	51	46.4 ± 12.6	43/8	29.4 ± 3.69	41.1 ± 3.4	4	45.3 ± 23.9	13.2 ± 21.1	NA	11.1 ± 4.7	80.7 ± 9.1
Zhang et al (2020)	RF + UPPP	30	37.00 ± 8.28	25/5	27.54 ± 3.98	38.97 ± 3.76	NA	48.1 ± 23.5	NA	NA	11.52 ± 5.37	70.3 ± 13.78
	CPAP Group	28	41.25 ± 9.17	25/3	28.82 ± 4.43	40.11 ± 2.67	NA	62.25 ± 17.11	NA	NA	13.46 ± 4.03	65.78 ± 15.71
	No treatment	32	37.78 ± 11.56	25/7	26.03 ± 3.46	37.85 ± 3.64	NA	42.91 ± 24.62	NA	NA	10.41 ± 5.06	76.75 ± 11.24
Abushab et al (2021)	Coblation + UPPP	12	44.83 ± 6.41	9/3	NA	NA	9	40.7 ± 16.09	NA	NA	13 ± 4.9	76.08 ± 18.64
Bosco et al (2021)	Coblation + ESP	24	49.16 ± 9.07	22/2	27.26 ± 2.78	NA	18	33.01 ± 17.53	NA	NA	11 ± 5.11	82.4 ± 10.3
Lu et al (2022)	Coblation + UPPP	12	34.9 ± 3.2	12/0	27.0 ± 1.9	NA	NA	43.8 ± 16.9	28.4 ± 15.6	NA	12.5 ± 5.1	NA
Duan et al (2023)	Coblation (CELL) + UPPP	26	46.1 ± 8.7	24/2	28.5 ± 3.4	NA	20	51.5 ± 18.9	NA	8.2 ± 1.1	14.7 ± 2.2	67.3 ± 10.1
	UPPP	30	44.8 ± 10.2	29/1	28.3 ± 3.8	NA	12	51.7 ± 15.8	NA	8.5 ± 1	14.3 ± 2.4	67.8 ± 8.2
Woodson et al (2023)	Placebo	30	46.0 ± 8.1	21/9	28.5 ± 4.2	40.6 ± 3.6	NA	15.4 ± 7.8	3.9 ± 4.1	0.37 ± 0.78	11.6 ± 3.5	NA
	RF	29	49.4 ± 9.2	26/3	27.7 ± 3.6	40.9 ± 3.3	NA	21.3 ± 11.1	7.5 ± 10.9	0.64 ± 1.46	11.9 ± 4.6	NA
	CPAP	28	51.7 ± 8.6	22/6	29.1 ± 3.7	41.4 ± 3.3	NA	19.8 ± 9.9	6.2 ± 7.5	NA	12.6 ± 5	NA

Abbreviations: CELL, coblation endoscopic lingual lightening; CPAP, continuous positive airway pressure; ESP, expansion sphincter pharyngoplasty; NA, not available; RF, radiofrequency; SMILE, submucosal minimally invasive lingual excision; UPPP, uvulopalatopharyngoplasty; ZPPP: z‐palatopharyngoplasty.

^a^ Ventral approach.

^b^ Dorsal approach.

Various complications were reported across the included studies. The primary postoperative concerns were dysphagia (7%, reported in 5 studies), respiratory distress due to edema (4%, reported in 2 studies), and postoperative bleeding (3%, reported in 3 studies). Intraoperative complications included bleeding (3%, reported in 11 studies) and hypoglossal nerve paralysis (2%, reported in 12 studies). Additional postoperative complications observed were taste sensation loss (6%, reported in 9 studies), aspiration (6%, reported in 2 studies), hematomas (4%, reported in 4 studies), and velopharyngeal insufficiency (16%, reported in 5 studies). **Table** 2 illustrates the complication rates associated with each intervention.

**Table 2:** Complication Rates of Included Interventions

	RF + UPPP		Coblation + UPPP		RF alone	
Complication	Rate (%)	Number of studies	Rate (%)	Number of studies	Rate (%)	Number of studies
Airway obstruction	0%	1	0%	0	0%	0
Intraoperative bleeding	0%	0	2%	4	0%	0
Tongue base abscess	0%	3	0%	2	0%	2
Edema leading to respiratory distress	0%	1	8%	3	0%	0
Dysphagia	0%	1	6%	4	10%	2
Speech impairment	5%	2	0%	3	0%	1
Nerve paralysis/hypoglossal nerve/neural damage	2%	5	2%	7	0%	2
transient loss of taste sensation/change	1%	1	13%	8	0%	1
Aspiration	0%	0	4%	2	7%	1
Postoperative bleeding	2%	3	5%	8	0%	1
Transient globus pharyngeus	0%	1	6%	1	0%	0
Transient tongue numbness	0%	1	2%	1	0%	0
Hematomas	0%	2	0%	0	8%	2
Oropharyngeal ulcerations	3%	5	0%	0	13%	2
Infections/tongue infection	0%	4	0%	0	1%	3
Postoperative intubation or tracheostomy	0%	0	0%	1	0%	0
Abscesses	0%	3	0%	2	0%	0
Velopharyngeal insufficiency (VPI)	12%	3	33%	2	0%	0

### Summary of the Quality Assessment

The results of observational studies are presented in **Table** 3. There were a total of 16 observational studies, of which 9 were rated as good quality and 7 as fair quality. The main limitations of the cohort studies were the lack of representativeness of the exposed cohort, the selection of the nonexposed cohort, and the adequacy of follow‐up. Only 3 cohort studies achieved 2 stars for comparability, indicating that they adjusted for the main confounders or used matching methods. The mean follow‐up duration ranged from 1 to 48 months, with a median of 6 months. There was only 1 case‐control study that had a good quality score. It had a valid case definition, a representative and well‐defined sample, and a comparable exposure assessment. The nonresponse rate was unknown. The results of the ROBINS‐I assessment are presented in **Table** 4. Out of the 19 studies, only 3 studies (Duan 2023; MacKay 2013; Eun 2009) were rated as having low overall risk of bias. Two studies (Abushab 2021; Nelson 2001) were rated as having a serious overall risk of bias, mainly due to confounding. The remaining 14 studies were rated as having a moderate overall risk of bias, with varying degrees of bias across different domains. The most common source of bias was bias due to confounding.

**Table 3:** Summary of Quality Assessment of Observational Studies

Cohort studies												
Baseline				Selection				Comparability	Outcome			
Study ID	Study design	Mean follow‐up	Sample size (n)	Representativeness of the exposed cohort	Selection of the nonexposed cohort	Ascertainment of exposure	Demonstration that outcome of interest was not present at the start of the study	Comparability of cohorts based on the design or analysis	Assessment of outcome	Was follow‐up long enough for outcomes to occur	Adequacy of follow‐up of cohorts	Quality Score
Stuck (2002)	Prospective	1 mo	20	*	‐	*	*	‐	*	‐	*	Fair
Friedman (2003)	Retrospective	6 mo	277	*	*	*	*	‐	*	*	‐	Good
Steward (2005)	Prospective	11 mo	29	*	‐	*	*	‐	*	*	*	Good
Blumen (2006)	Prospective	6 mo	11	*	‐	*	*	‐	*	*	*	Good
Friedman (2007)	Retrospective	6 mo	122	*	‐	*	*	‐	*	*	‐	Fair
Eun (2008)	Prospective	6 mo	66	*	‐	*	*	‐	*	*	‐	Fair
Friedman (2008)	Retrospective	3 mo	96	*	*	*	*	**	*	*	*	Good
Babademez (2010)	Retrospective	6 mo	16	‐	‐	*	*	‐	*	*	*	Fair
Hou (2012)	Prospective	12 mo	40	*	*	*	*	**	*	*	*	Good
MacKay (2013)	Prospective	36 mo	48	*	*	*	*	‐	*	*	*	Good
Lin (2015)	Retrospective	3 mo	35	*	*	*	*	‐	*	*	*	Good
Chen (2019)	Prospective	24 mo	22	*	‐	*	*	‐	*	*	*	Fair
Bahgat (2020)	Retrospective	3 mo	25	‐	‐	*	*	‐	*	*	*	Fair
Bosco (2021)	Prospective	12 mo	24	‐	‐	*	*	‐	*	*	*	Fair
Lu (2022)	Prospective	3 mo	12	*	‐	*	*	‐	*	*	*	Good

Case‐control studies												
Baseline				Selection				Comparability	Outcome			
Study ID	Study design	Mean follow‐up	Sample size (n)	Is the case definition adequate?	Representativeness of the cases	Selection of controls	Definition of controls	Comparability of cases and controls on the basis of the design or analysis	Ascertainment of exposure	Same method of ascertainment for cases and controls	Nonresponse rate	Quality score
Li (2016)	Retrospective	6 mo	90	*	‐	*	*	*	*	*	*	Good

**Table 4:** Summary of Quality Assessment of Nonrandomized Studies of Interventions (NRSI)

Study ID	D1: bias due to confounding	D2: bias in selection of participants into the study	D3: bias in classification of interventions	D4: bias due to deviations from intended interventions	D5: bias due to missing data	D6: bias in measurement of outcomes	D7: Bias in selection of the reported result	Overall
Nelson (2001)	Serious	Low	Low	Low	Low	Low	Low	Serious
Riley (2003)	Moderate	Low	Low	Low	Low	Low	Low	Moderate
Stuck (2004)	Moderate	Low	Low	Low	Low	Low	Low	Moderate
Steward (2005)	Moderate	Low	Low	Low	Moderate	Low	Low	Moderate
Herder (2006)	Moderate	Low	Low	Low	Low	Low	Low	Moderate
Richard (2006)	Moderate	Low	Low	Low	Low	Low	Low	Moderate
Welt (2006)	Moderate	Low	Low	Low	Low	Low	Low	Moderate
Tvinnereim (2007)	Moderate	Low	Low	Low	Low	Low	Low	Moderate
Ceylan (2009)	Moderate	Moderate	Moderate	Moderate	Moderate	Low	Low	Moderate
Eun (2009)	Low	Low	Low	Low	Low	Low	Low	Low
Neruntarat (2009)	Moderate	Low	Low	Low	Moderate	Low	Low	Moderate
Eun (2013)	Moderate	Low	Low	Low	Low	Low	Low	Moderate
MacKay (2013)	Low	Low	Low	Low	Low	Low	Low	Low
Plzak (2013)	Moderate	Low	Low	Low	Low	Low	Low	Moderate
Wang (2013)	Moderate	Low	Low	Low	Low	Low	Low	Moderate
Li (2015)	Moderate	Low	Low	Low	Low	Low	Low	Moderate
Zhang (2020)	Moderate	Low	Low	Low	Low	Low	Low	Moderate
Abushab (2021)	Serious	Low	Low	Low	Low	Moderate	Low	Serious
Duan (2023)	Low	Low	Low	Low	Low	Low	Low	Low

**Figure 2:**
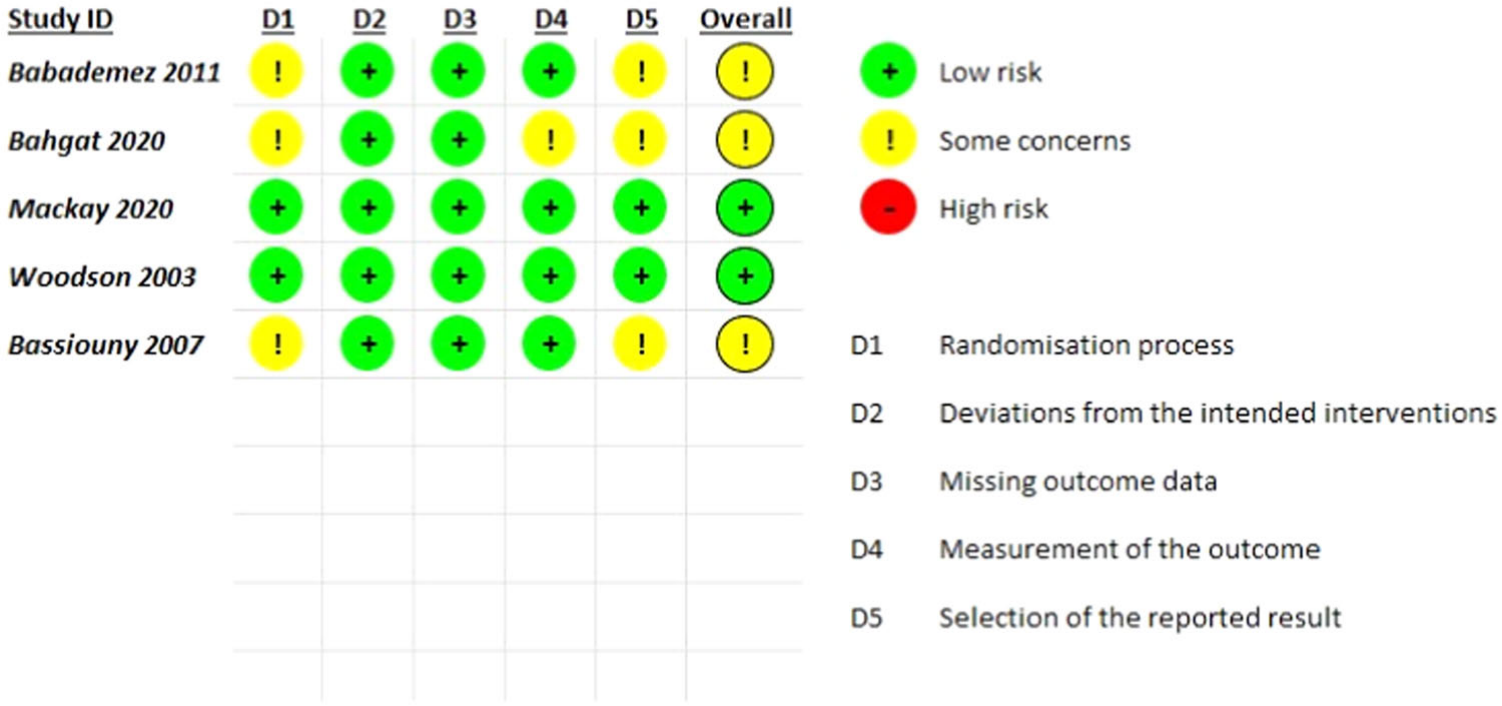
Summary of quality assessment of randomized‐controlled trials.

The results of the ROB‐2 assessment are demonstrated in **Figure** 2. Out of 5 RCTs, only 2 studies (Woodson 2003; Mackay 2020) were judged to have a low risk of bias overall, while 3 studies (Babademez 2011; Bassiouny 2007; Bahgat 2020) had some concerns across all domains. The main sources of bias were related to the randomization process, deviations from the intended interventions, missing outcome data, and selection of the reported result. Results of Egger's test for publication bias are illustrated in **Table** 5.

**Table 5:** Summary of Egger's Test for Publication Bias

	Intercept	*P* value
**BMI**	−1.42	.03
**AHI**	−0.07	.93
**AI**	0.22	.81
**VAS**	−0.35	.52
**ESS**	−0.25	.31
**SpO_2_**	−0.28	.17

### Meta‐analysis of the Endpoints

#### Direct Meta‐Analysis

Overall, there were only a small number of direct comparisons across the studies, making it difficult to draw meaningful conclusions in terms of comparing 2 arms against each other. The main comparisons were (1) coblation + UPPP versus UPPP only, (2) RF + UPPP versus UPPP only, and (3) RF + UPPP versus coblation + UPPP. A direct comparison of the different treatment arms was not possible to be conducted for surgical success rate due to an insufficient number of studies.

### Primary Outcomes of Direct Meta‐Analysis

#### AHI

**Figure 3:**
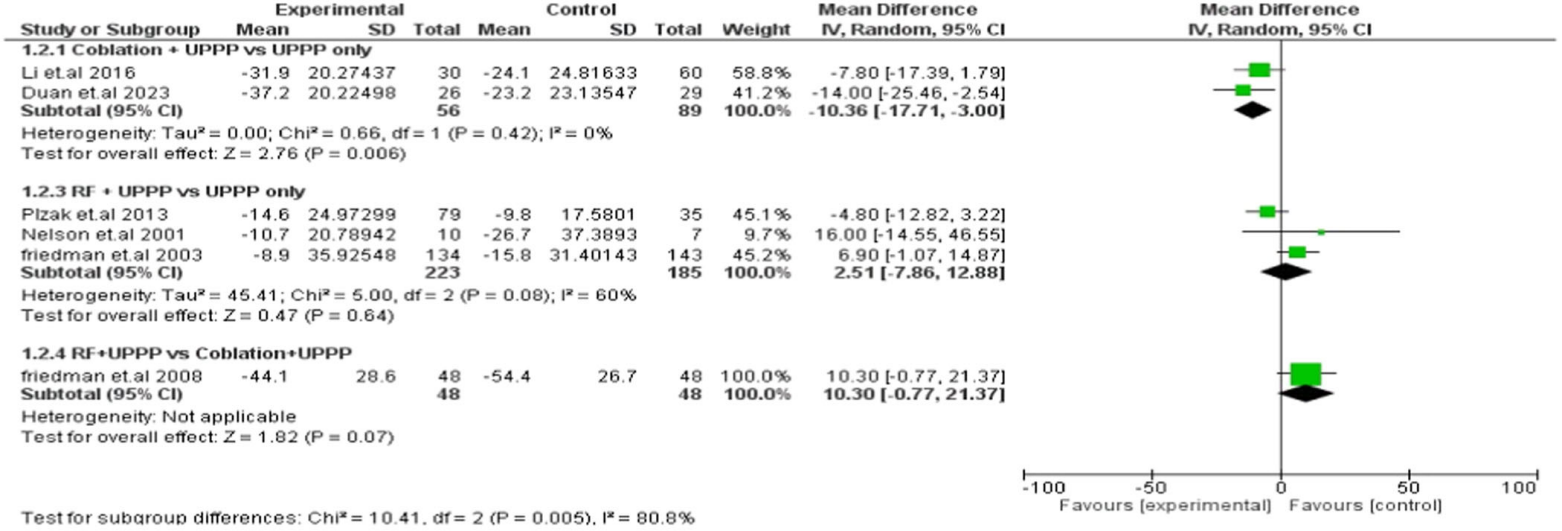
Forest plot for mean difference analysis between different interventions—apnea–hypopnea index (AHI).

Coblation + UPPP was associated with a statistically significant reduction in AHI compared to UPPP only (MD: −10.36, 95% CI [17.71, −3.00], *I* ^2^ = 0%). In contrast, there was no significant difference between RF + UPPP and UPPP only (MD: 2.51, 95% CI [−7.86, 12.88], *I* ^2^ = 60%) or when comparing RF + UPPP and coblation + UPPP (MD: 10.30, 95% CI [−0.77, 21.37]), as illustrated in **Figure** 3.

#### AI and ESS

**Figure 4:**
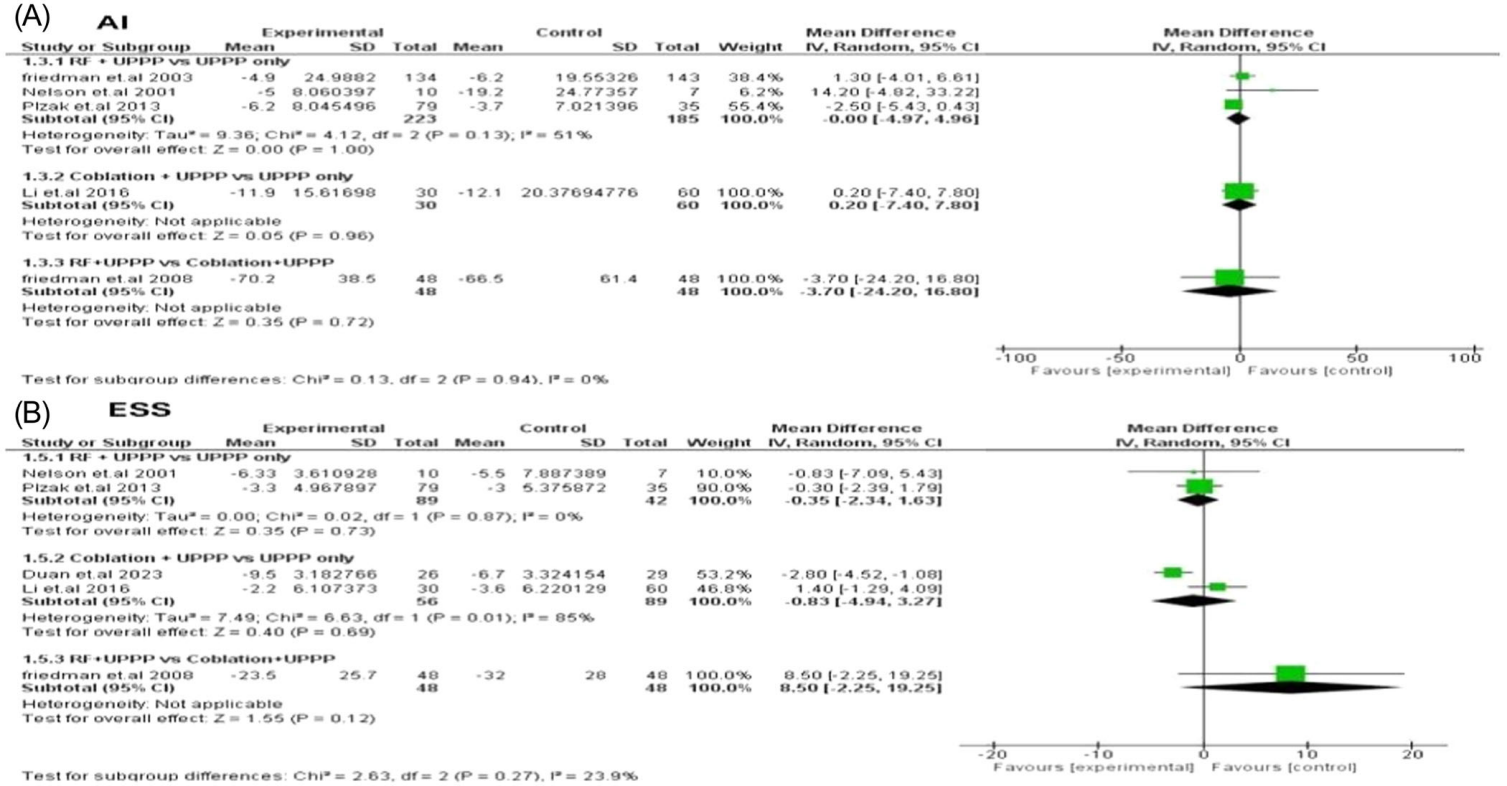
Forest plot for mean difference analysis between different interventions—(A) apnea index (AI); (B) Epworth sleepiness score (ESS).

For other primary outcomes such as ESS and AI, there were no significant differences between any of the different arms in any of the 3 comparisons, as demonstrated in **Figure** 4.

#### Secondary Outcomes of Direct Meta‐Analysis

For secondary outcomes that include VAS, SpO_2_, and BMI, there were no statistically significant differences across any of the different arms in any of the comparisons across all groups.

### Single‐Arm Meta‐Analysis

#### Primary Outcomes of Single‐Arm Meta‐Analysis

##### Surgical Success Rate

**Figure 5:**
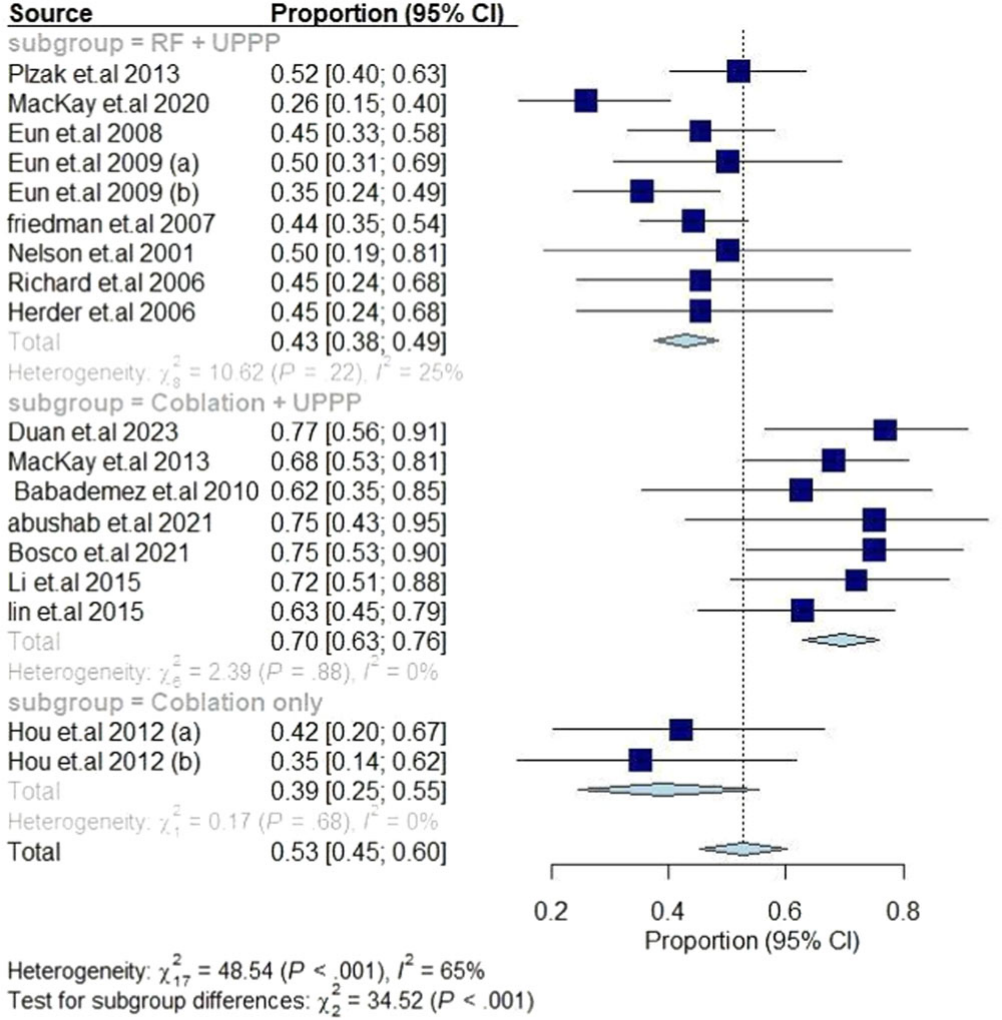
Mean change in surgical success rate among different interventions.

Regarding surgical success rate as defined by Sher criteria, the greatest percentage of success was exhibited in the coblation + UPPP subgroup with a 70% success rate (95% CI [63%, 76%], *I* ^2^ = 0%), followed by RF + UPPP with 43% success rate (95% CI [38%, 49%], *I* ^2^ = 25%), and coblation only with a success rate of 53% (95% CI [25%, 55%], *I* ^2^ = 0%), as depicted in **Figure** 5.

##### AHI

**Figure 6:**
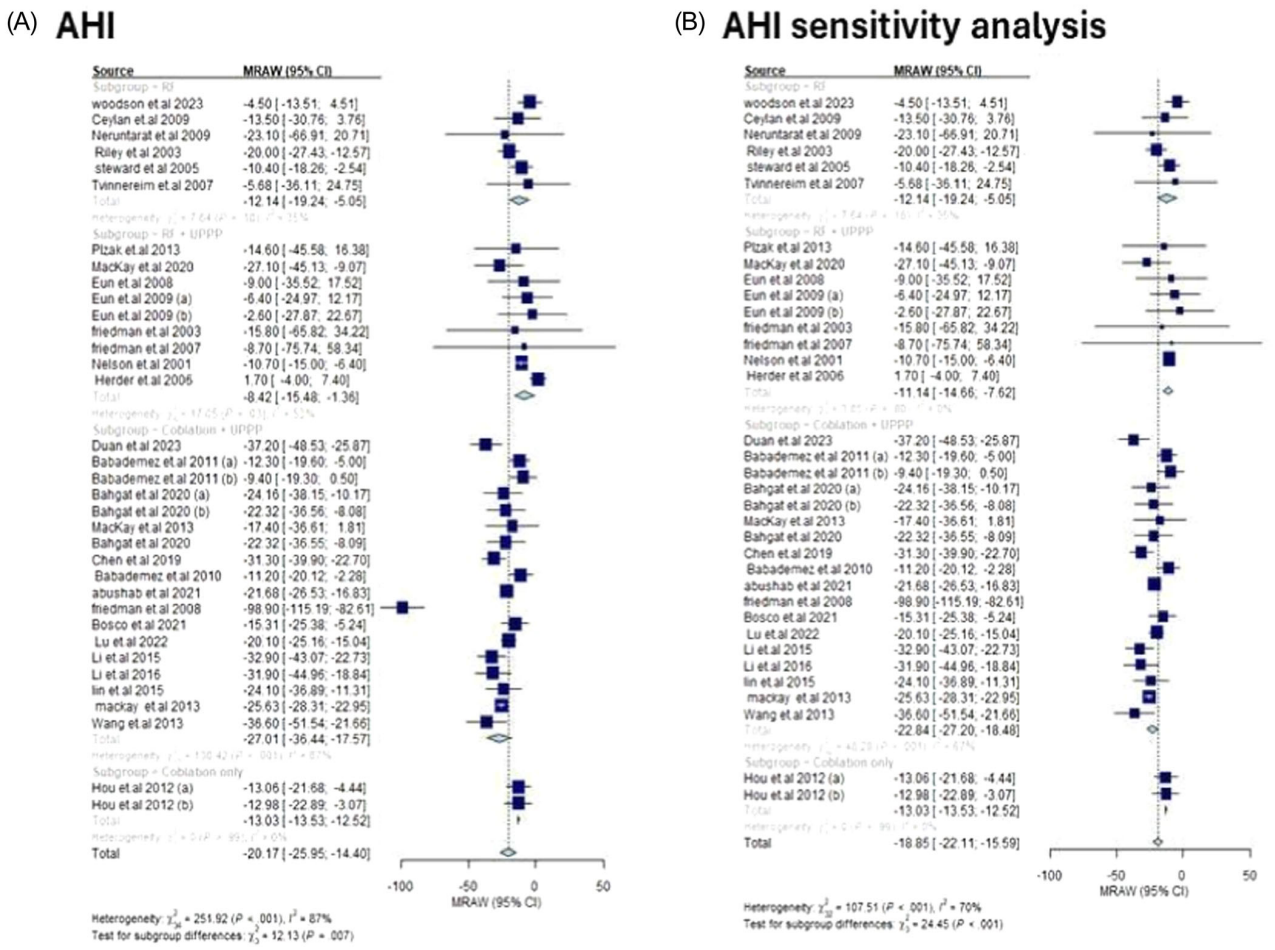
(A) Mean change in AHI among different interventions. (B) Mean change in AHI among different interventions after sensitivity analysis. AHI, apnea–hypopnea index.

**Figure** 6 demonstrates that the greatest reduction in AHI was exhibited in the coblation + UPPP subgroup (MD: −22.84, 95% CI [−27.20, −18.48], *I* ^2^ = 67%), followed by coblation only (MD: −13.03, 95% CI [−13.53, −12.52], *I* ^2^ = 0%), RF only (MD: −12.14, 95% CI [−19.24, −5.05], *I* ^2^ = 35%), and RF + UPPP (MD: −11.14, 95% CI [−14.66, −7.62], *I* ^2^ = 0%).

To resolve heterogeneity in the RF + UPPP and coblation + UPPP subgroups, the study of Friedman et al (2008) was excluded. The estimate for RF + UPPP changed to (MD: −11.14, 95% CI [−14.66, −7.62], *I* ^2^ = 0%) and coblation + UPPP (MD: −22.84, 95% CI [−27.20, −18.48], *I* ^2^ = 67%), with coblation + UPPP remaining the intervention associated with the greatest change.

##### AI

Consistent with AHI, coblation + UPPP led to the greatest change in the score (MD: −17.99, 95% CI [−21.17, −4.80], *I* ^2^ = 64%), followed by RF + UPPP (MD: −15.88, 95% CI [−35.08, 3.31], *I* ^2^ = 87%) and RF (MD: −6.69, 95% CI [−14.05, 0.67], *I* ^2^ = 83%).

To resolve the heterogeneity in all subgroups, the study of Blumen et al (2006) was removed from the RF subgroup, the study of Friedman et al was removed from the RF + UPPP, and the study of Chen et al (2019) was removed from the coblation + UPPP.

**Figure 7:**
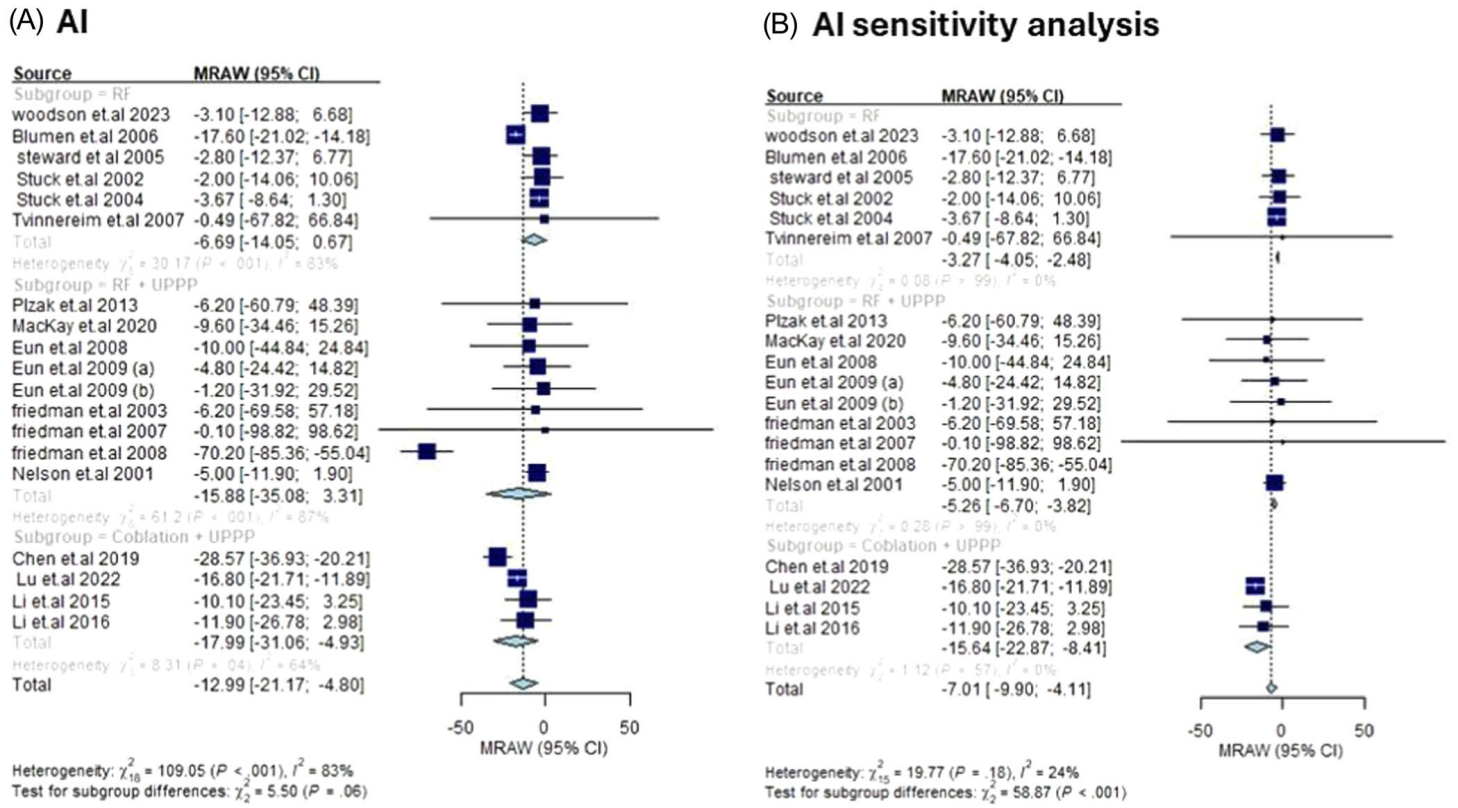
(A) Mean change in AI among different interventions. (B) Mean change in AI among different interventions after sensitivity analysis. AI, apnea index.

Across the 3 subgroups, the heterogeneity was resolved (*I* ^2^ = 0%), and coblation + UPPP remained associated with the highest change (MD: −15.64, 95% CI [−22.87, −8.41]), followed by RF + UPPP (MD: −5.26, 95% CI [−6.70, −3.82]), and RF (MD: −3.27, 95% CI [−4.05, −2.48]), as shown in **Figure** 7.

##### ESS

Coblation + UPPP (MD: −7.59, 95% CI [−13.58, −1.61], *I* ^2^ = 38%) was associated with greater change in the score compared to RF + UPPP (MD: −7.18, 95% CI [−11.69, −2.68], *I* ^2^ = 0%). Although there was no significant heterogeneity, the study of Friedman et al (2008) had a much greater change in the score compared to the other studies, and we believed that this might have led to a biased score change. Therefore, a sensitivity analysis was performed by removing it.

This led to reduction in the change in the coblation + UPPP (MD: −4.72, 95% CI [−5.94, −23.31]) and RF + UPPP (MD: −4.82, 95% CI [−6.08, −3.57]), as demonstrated in Supplemental Figures S1 and S2, available online.

### Secondary Outcomes of Single‐Arm Meta‐Analysis

#### BMI

RF was associated with an increase in BMI (MD: 0.22, 95% CI [0.08, 0.35], *I* ^2^ = 0%), while RF + UPPP (MD: −4.09, 95% CI [−19.05, 10.86], *I* ^2^ = 85%) and coblation + UPPP (MD: −0.35, 95% CI [−0.69, −0.01], *I* ^2^ = 0%) were associated with reductions in BMI. To resolve heterogeneity in the RF + UPPP arm, the study of Richard et al (2006) was excluded, and the final estimate was changed to be MD: 0.12, 95% CI [−0.28, 0.52], *I* ^2^ = 0%, as demonstrated in Supplemental Figures S3 and S4, available online.

#### VAS

For VAS, the greatest reduction was seen in the RF + UPPP subgroup (MD: −18.21, 95% CI [−40.40, 3.98], *I* ^2^ = 74%), followed by coblation + UPPP (MD: −16.20, 95% CI [−41.81, 9.42], *I* ^2^ = 88%), and RF only (MD: −2.68, 95% CI [−3.57, −1.78], *I* ^2^ = 0%).

However, like other outcomes, Friedman et al (2008) was introducing heterogeneity in both RF + UPPP and coblation + UPPP subgroups and after excluding this study, the heterogeneity was resolved in both subgroups and coblation + UPPP (MD: −5.73, 95% CI [−6.41, −5.05]) became associated with greater reduction in the VAS score compared to RF + UPPP (MD: −4.73, 95% CI [−5.32, −4.15]), which is shown in Supplemental Figures S5 and S6, available online.

#### SpO_2_

Supplemental Figure S7, available online illustrates the mean increase in SpO_2_ among different interventions. Coblation + UPPP (MD: 7.43, 95% CI [4.94, 9.91], *I* ^2^ = 0%) had the highest increase in oxygen saturation, followed by RF only (MD: 9.93, 95% CI [4.64, 15.22], *I* ^2^ = 0%) and RF + UPPP (MD: 4.25, 95% CI [1.26, 7.24], *I* ^2^ = 0%).

## Discussion

The current meta‐analysis evaluated the effectiveness and safety profile of coblation versus radiofrequency for BOT reduction in OSA patients. The analysis included 40 studies, comprising 1940 patients, with 1440 patients undergoing BOT reduction interventions (RF = 306, RF + UPPP = 656, and coblation + UPPP = 482). However, only a limited number of studies were incorporated into the direct meta‐analysis due to the scarcity of head‐to‐head comparisons between the different interventions. The remaining studies were single‐arm studies, and outcomes were quantitatively analyzed and reported. The overall quality assessment demonstrated low risk of bias in 14 studies, some concerns in 24 studies, and high risk of bias in 2 studies. The pooled results demonstrated that coblation + UPPP was superior to both RF + UPPP and RF alone in addressing BOT hypertrophy, based on primary outcomes such as surgical success rate (defined by Sher criteria), mean reduction in AHI, and AI postoperatively. All interventions were comparable in terms of ESS, VAS, BMI, and SpO_2_. When comparing single‐arm trials of coblation + UPPP, RF + UPPP, and RF alone, surgical success rate was much higher in coblation + UPPP (70%) compared to RF + UPPP (43%) and RF (40%). In addition, coblation + UPPP was associated with the greatest reduction in AHI and AI postoperatively at −22.84 and −15.64, respectively, compared to RF + UPPP at −11.14 and −5.26, respectively. Complication rates were comparable between interventions.

The increasing volume of research on BOT hypertrophy interventions for OSA patients highlights the necessity to identify the most effective and safe approach. Vicini et al^19^ introduced transoral robotic surgery (TORS) in 2010 for BOT hypertrophy and lingual tonsil resection in OSA patients, demonstrating promising functional results, including improved quality of life, enhanced swallowing, and manageable pain. TORS offers significant advantages in terms of superior visualization and tissue manipulation. Other effective techniques include radiofrequency ablation (RFBOT) and CTBR, with CTBR showing comparable outcomes to TORS for BOT hypertrophy.^4^, ^20^

Our findings that coblation + UPPP is superior to RF + UPPP and RF alone align with the observations of Lee et al^5^ who noted similar magnitudes of reduction in AHI compared to TORS and plasma ablation, another minimally invasive technique for BOT reduction. Specifically, Lee et al^5^ reported postoperative weighted MDs in AHI of –23.92 for TORS and –22.07 for plasma ablation, with surgical success rates of 57.6% and 60.3%, respectively, with no statistically significant difference between the 2 groups (*P* = .4474). Moreover, the postoperative weighted MDs of AHI in the TORS and PA groups were –23.92 and –22.07, respectively, with each demonstrating significant overall effect (*P *< .00001). Postoperative bleeding was significantly lower in TORS (3.3%) compared to PA (7.5%) (*P* = .0103). Furthermore, the present study also supports the findings of Calvo‐Henriquez et al^20^ who reported higher overall complication rates in coblation (44.42%) compared to TORS (35.78%), but with comparable effectiveness in reducing AHI and improving surgical success rates. The superiority of coblation + UPPP in our meta‐analysis, particularly in terms of surgical success rate and reduction in AHI and AI, reproduces the findings of Cammaroto et al^21^ who observed no significant differences in clinical parameters between TORS and coblation, but noted slightly lower complication rates with coblation (8.4% vs 21.3%). This consistency across studies underscores the robustness of coblation as an effective intervention for BOT reduction in OSA patients.

The findings of the current systematic review and meta‐analysis have substantial clinical implications. The superiority of coblation + UPPP in terms of surgical success rate (70% vs 43% for RF + UPPP and 40% for RF alone) and reductions in AHI (–22.84 vs –11.14) and AI (–15.64 vs –5.26) postoperatively suggests that it may be a more effective intervention for BOT hypertrophy in OSA patients. This aligns with previous findings by Cammaroto et al^21^ who noted comparable effectiveness between coblation and TORS, but with a lower complication rate for coblation (8.4% vs 21.3%). Similarly, Lee et al^5^ found that both TORS and plasma ablation achieved significant reductions in AHI, with comparable surgical success rates (57.6% for TORS and 60.3% for plasma ablation), supporting the effectiveness of minimally invasive techniques for BOT reduction. In contrast, Calvo‐Henriquez et al^20^ revealed that the highest overall complication rate was with coblation at 44.42% followed by TORS at 35.78% and only 4.4% in RFBOT. The most reported complication overall was infection (1.95%), followed by transient dysphagia (1.30%), severe mouth floor edema (0.84%), and permanent taste disorder (0.47%).^20^ However, the current study highlights the need for caution due to the lack of direct head‐to‐head comparisons between coblation and RF. Further well‐designed, high‐quality research is necessary to consolidate these findings and provide stronger evidence for clinical decision‐making. The results of this meta‐analysis, despite its limitations, contribute valuable insights into the effectiveness and safety of BOT reduction techniques and underscore the need for ongoing research to optimize treatment strategies for OSA patients.

These findings may be particularly relevant in global contexts where alternative treatments such as HGNS are not widely accessible, emphasizing the importance of options such as RF and coblation for BOT surgeries.

While this meta‐analysis provides valuable insights, the limited number of direct comparisons between RF and coblation remains a critical limitation. Future studies should aim for direct comparisons to provide more robust evidence on the relative efficacy of these interventions.

### Strengths and Limitations

This meta‐analysis represents the first comprehensive investigation into the effectiveness and safety profiles of CBTR and RFBOT reduction for the treatment of OSA. A notable strength is the inclusion of both direct and single‐arm meta‐analyses, which allows for a more nuanced understanding of the potential superiority of one technique over the other. However, the study is not without its limitations. Most of the included studies were prospective and retrospective cohort studies, with only 4 RCTs, which may introduce selection and reporting biases. The absence of well‐designed direct comparisons between CBTR and RFBOT significantly limits the ability to generalize the conclusions. Additionally, discrepancies in the associated procedures performed alongside CBTR and RFBOT, as well as the heterogeneity in patient selection and technique application, further complicate the generalizability of the results. The findings from tongue channeling should not be directly compared to tongue base reduction, as these are fundamentally different interventions. Despite these limitations, the current data identify critical gaps in the literature and serves as a foundation for guiding future research.

## Conclusion

The current study illustrates significant differences between coblation and radiofrequency for patients undergoing BOT reduction, either alone or as part of multilevel sleep surgery for the treatment of OSA, particularly in terms of surgical success rates and mean reductions in AHI and AI postoperatively. Coblation demonstrated superiority over radiofrequency in these outcomes. However, due to the lack of direct head‐to‐head comparisons, conclusions drawn from this study should be interpreted with caution. Additional RCTs are essential to strengthen the evidence base, provide more definitive comparisons, and demonstrate long‐term effectiveness. Such studies would also help identify prognostic characteristics that could lead to improved surgical success and more personalized treatment strategies for OSA patients.

## Author Contributions

**Salman Hussain**, conception of idea, data collection, data analysis, manuscript drafting, and revision; **Jafar Hayat**, data analysis, manuscript drafting, and revision; **Raisa Chowdhury**, manuscript drafting and revision; **Mahmoud Ibrahim**, manuscript drafting; **Abdulmohsen AlTerki**, manuscript revision; **Ahmed Bahgat**, manuscript drafting and revision; **Ahmed A. AlSayed**, manuscript drafting; **Vikram Padhye**, manuscript drafting; **Robson Capasso**, manuscript revision.

## Disclosures

### Competing interests

None.

### Funding source

None.

## Supporting information

Supporting information.

Supporting information.

Supporting information.

Supporting information.
