# Supplementary figures and images for: Coblation Versus Radiofrequency for Tongue Base Reduction in Obstructive Sleep Apnea: A Meta‐analysis
Source: OTO Open. 2025 Jan 19;9(1):e70076. doi: 10.1002/oto2.70076 (PMC11743998; doi:10.1002/oto2.70076)

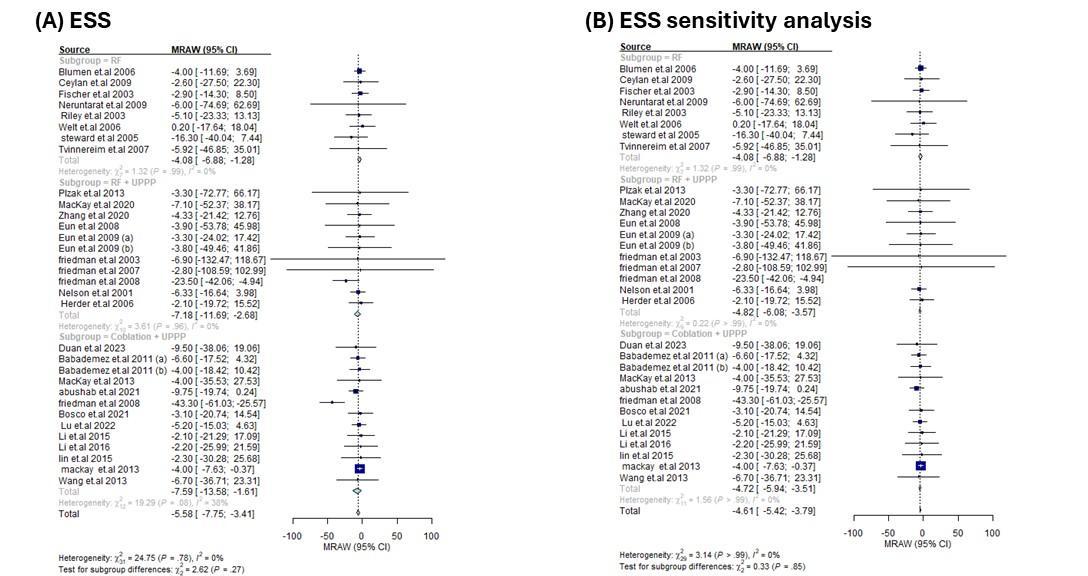

Supplement: Supplementary file 1 — Supporting information. [file OTO2-9-e70076-s002.docx]

**
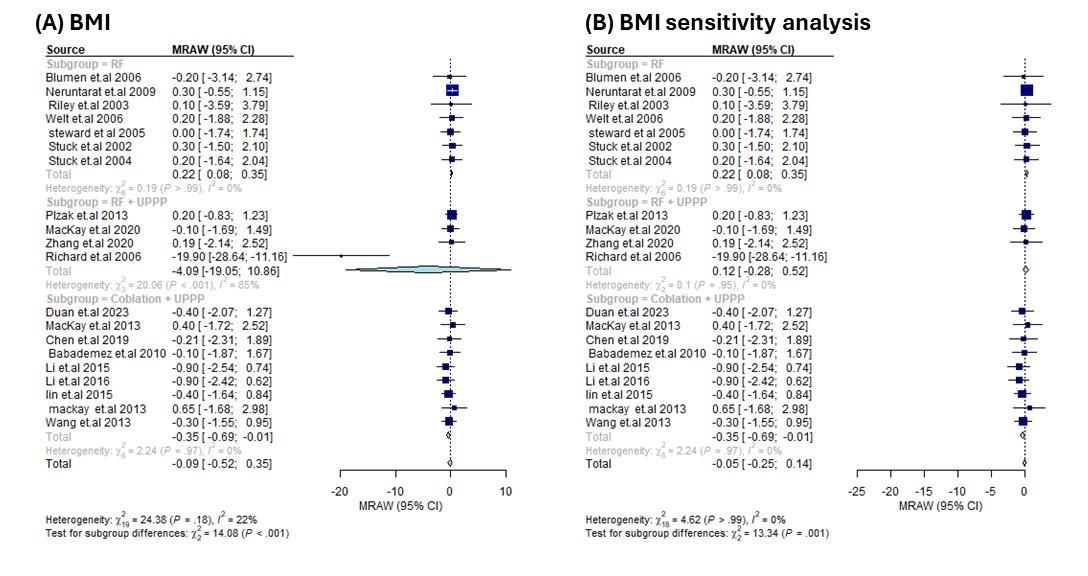
**

Supplement: Supplementary file 2 — Supporting information. [file OTO2-9-e70076-s003.docx]

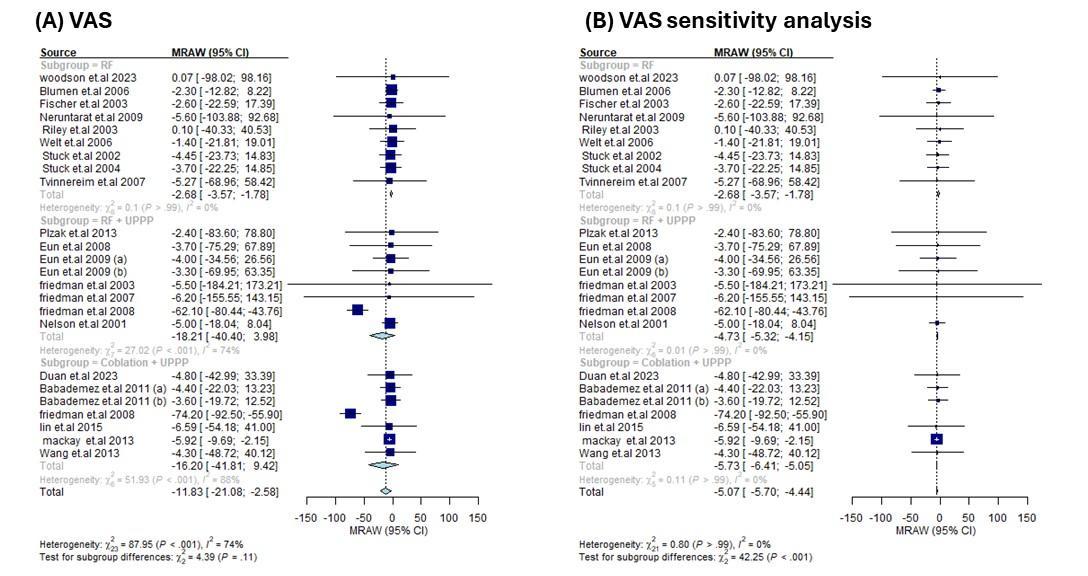

Supplement: Supplementary file 3 — Supporting information. [file OTO2-9-e70076-s004.docx]

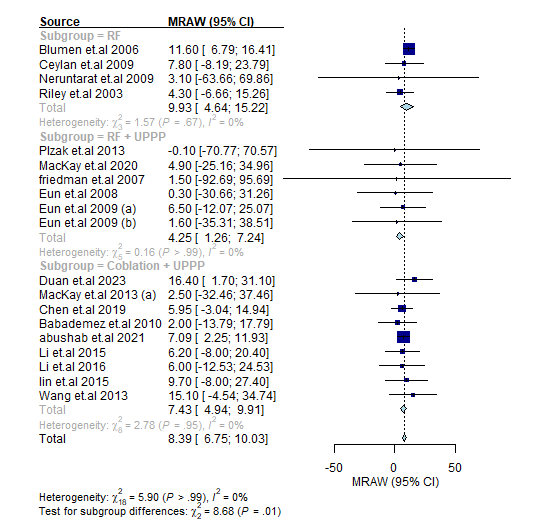

Supplement: Supplementary file 4 — Supporting information. [file OTO2-9-e70076-s001.docx]
